# Tumor and circulating biomarkers in patients with second-line hepatocellular carcinoma from the randomized phase II study with tivantinib

**DOI:** 10.18632/oncotarget.11621

**Published:** 2016-08-25

**Authors:** Lorenza Rimassa, Giovanni Abbadessa, Nicola Personeni, Camillo Porta, Ivan Borbath, Bruno Daniele, Stefania Salvagni, Jean-Luc Van Laethem, Hans Van Vlierberghe, Jörg Trojan, Enrico N. De Toni, Alan Weiss, Steven Miles, Antonio Gasbarrini, Monica Lencioni, Maria E. Lamar, Yunxia Wang, Dale Shuster, Brian E. Schwartz, Armando Santoro

**Affiliations:** ^1^ Humanitas Cancer Center, Humanitas Clinical and Research Center, Rozzano, Milan, Italy; ^2^ ArQule, Burlington, MA, USA; ^3^ Department of Medical Biotechnology and Translational Medicine, University of Milan, Milan, Milan, Italy; ^4^ Fondazione IRCCS Policlinico San Matteo, Pavia, Italy; ^5^ Cliniques Universitaires Saint-Luc, Brussels, Belgium; ^6^ G Rummo Hospital, Benevento, Italy; ^7^ Azienda Ospedaliera Universitaria Sant'Orsola-Malpighi, Bologna, Italy; ^8^ Erasme University Hospital, Brussels, Belgium; ^9^ Ghent University Hospital, Ghent, Belgium; ^10^ J W Goethe-University Hospital, Frankfurt, Germany; ^11^ Klinikum der Universitaet Muenchen-Grosshadern, Munich, Germany; ^12^ Vancouver General Hospital, British Columbia Cancer Clinic, Vancouver, Canada; ^13^ Cedar Sinai, Los Angeles, CA, USA; ^14^ Policlinico Universitario Agostino Gemelli, Rome, Italy; ^15^ Azienda Ospedaliero-Universitaria di Pisa, Pisa, Italy; ^16^ Daiichi Sankyo, Edison, NJ, USA; ^17^ Humanitas University, Rozzano, Milan, Italy

**Keywords:** HCC, MET, HGF, AFP, sorafenib

## Abstract

ARQ 197-215 was a randomized placebo-controlled phase II study testing the MET inhibitor tivantinib in second-line hepatocellular carcinoma (HCC) patients. It identified tumor MET as a key biomarker in HCC.

Aim of this research was to study the prognostic and predictive value of tumor (MET, the receptor tyrosine kinase encoded by the homonymous *MNNG-HOS* transforming gene) and circulating (MET, hepatocyte growth factor [HGF], alpha-fetoprotein [AFP], vascular endothelial growth factor [VEGF]) biomarkers in second-line HCC. Tumor MET-High status was centrally assessed by immunohistochemistry. Circulating biomarkers were centrally analyzed on serum samples collected at baseline and every 4-8 weeks, using medians as cut-off to determine High/Low status. Tumor MET, tested in 77 patients, was more frequently High after (82%) versus before (40%) sorafenib. A significant interaction (*p* = 0.04) between tivantinib and baseline tumor MET in terms of survival was observed. Baseline circulating MET and HGF (102 patients) High status correlated with shorter survival (HR 0.61, *p* = 0.03, and HR 0.60, *p* = 0.02, respectively), while the association between AFP (104 patients) or VEGF (103 patients) status and survival was non-significant.

Conclusions: Tumor MET levels were higher in patients treated with sorafenib. Circulating biomarkers such as MET and HGF may be prognostic in second-line HCC. These results need to be confirmed in larger randomized clinical trials.

## INTRODUCTION

Hepatocellular carcinoma (HCC) is among the leading causes of cancer-related death [1], and sorafenib is the only approved systemic agent for patients with unresectable disease [2, 3]. Identifying biomarkers that can predict treatment efficacy will help select additional therapeutic strategies [4, 5].

The *MNNG-HOS* transforming gene (*MET*) encoding the receptor tyrosine kinase for hepatocyte growth factor (HGF) is involved in cancer progression and metastasis formation, and suggests poor prognosis in early stage and second-line HCC patients [6-8]. Hypoxia and oxidative stress can trigger MET expression or activation in HCC, and MET is involved in the resistance to antiangiogenic therapy [6, 7, 9, 10], its expression increasing in HCC cells resistant to sorafenib [11, 12]. MET can even self-activate and be HGF independent [13]. MET is also involved in the immune system development and bone marrow maturation [14, 15].

Tivantinib is an oral, ATP-independent inhibitor of MET, binding to its inactive form [16, 17]; its anti-MET effect was confirmed by several groups, though some also suggested additional targets [18-20], including a weak non-specific anti-tubulin activity [21-23]. However, neurotoxicity, a hallmark of tubulin inhibitors, has not been reported in over 2000 patients treated with tivantinib to date, even at doses much higher than the therapeutic one [8, 24-28]. Therefore, tivantinib may have additional targets, though these have yet to be confirmed. Tumor MET levels have been shown to decrease in both mice and patients treated with tivantinib [17, 29]. In clinical trials, tivantinib showed significant activity in patients with MET-High tumors such as non-squamous non-small cell lung cancer (NSCLC), colorectal cancer, HCC, and androgen-resistant metastatic prostate cancer [8, 27, 28, 30, 31]. In contrast, a study in MET-Low breast cancer patients showed no response to tivantinib [32].

Following promising phase I study results with tivantinib as monotherapy and in combination with sorafenib in HCC [26, 33], the ARQ 197-215 randomized, placebo-controlled phase II study with tivantinib (2:1 randomization to tivantinib or placebo) was conducted in 107 HCC patients (71 on tivantinib, 36 on placebo) pretreated with systemic therapy. Informed consent in writing was obtained from each patient and the study protocol conformed to the ethical guidelines of the 1975 Declaration of Helsinki as reflected in a priori approval by the appropriate Institutional review committee. The study reached its primary endpoint of time to progression in the intent-to-treat population and all pre-specified secondary efficacy endpoints in MET-High patients. Exploratory endpoints included the relationship between biomarkers and key efficacy endpoints [8].

Considering the lack of accepted biomarkers in HCC and realizing the necessity of placebo-controlled studies, we analyzed the prognostic and predictive values of tumor (MET) and circulating (MET, HGF, alpha-fetoprotein (AFP), vascular endothelial growth factor (VEGF)) biomarkers in the ARQ 197-215 study.

## RESULTS

### Tumor MET

Tumor samples of sufficient quality for tumor MET analysis at immunohistochemistry (IHC) were submitted for a total of 77 patients, 49 who were randomized to tivantinib and 28 to placebo [8]. Patient baseline characteristics were generally balanced between groups (Table 1).

**Table 1:** Patient baseline characteristics according to biomarker status

	Tumor			Circulating											
	MET			MET			HGF			AFP			VEGF		
	High	Low	*p* value	High	Low	*p* value	High	Low	*p* value	High	Low	*p* value	High	Low	*p* value
Age, median (range)	71 (46-85)	66 (27-83)	0.13	67 (27-83)	69 (35-85)	0.24	71 (35-85)	66 (27-83)	0.05	69 (27-85)	70 (45-81)	0.67	69 (35-83)	69 (27-85)	0.5
Sex, N (%)			1.00			0.80			0.44			0.31			0.21
Female	6 (16.2)	7 (17.5)		10 (19.6)	8 (15.7)		11 (21.6)	7 (13.7)		12 (23.1)	7 (13.5)		7 (13.5)	12 (23.5)	
Male	31 (83.8)	33 (82.5)		41 (80.4)	43 (84.3)		40 (78.4)	44 (86.3)		40 (76.9)	45 (86.5)		45 (86.5)	39 (76.5)	
Race, N (%)			0.05			1.00			0.49			0.69			0.84
Caucasian	35 (94.6)	33 (82.5)		45 (88.2)	46 (90.2)		47 (92.2)	44 (86.3)		45 (86.5)	48 (92.3)		47 (90.4)	45 (88.2)	
Asian	2 (5.4)	1 (2.5)		2 (3.9)	3 (5.9)		2 (3.9)	3 (5.9)		3 (5.8)	2 (3.8)		2 (3.8)	3 (5.9)	
Black/African American	0 (0.0)	5 (12.5)		3 (5.9)	2 (3.9)		1 (2.0)	4 (7.8)		3 (5.8)	2 (3.8)		3 (5.8)	2 (3.9)	
ECOG PS, N (%)			0.65			0.23			0.02			0.17			0.02
0	22 (59.5)	26 (65.0)		25 (49.0)	32 (62.7)		22 (43.1)	35 (68.6)		25 (48.1)	33 (63.5)		23 (44.2)	35 (68.6)	
1	15 (40.5)	14 (35.0)		26 (51.0)	19 (37.3)		29 (56.9)	16 (31.4)		27 (51.9)	19 (36.5)		29 (55.8)	16 (31.4)	
Vascular invasion, N (%)			0.23			0.67			0.09			0.03			1.00
Yes	9 (24.3)	15 (37.5)		15 (29.4)	18 (35.3)		21 (41.2)	12 (23.5)		22 (42.3)	11 (21.2)		17 (32.7)	16 (31.4)	
No	28 (75.7)	25 (62.5)		36 (70.6)	33 (64.7)		30 (58.8)	39 (76.5)		30 (57.7)	41 (78.8)		35 (67.3)	35 (68.6)	
Distant metastases, N (%)			0.64			0.53			0.83			0.68			0.30
Yes	23 (62.2)	27 (67.5)		36 (70.6)	32 (62.7)		33 (64.7)	35 (68.6)		33 (63.5)	36 (69.2)		37 (71.2)	31 (60.8)	
No	14 (37.8)	13 (32.5)		15 (29.4)	19 (37.3)		18 (35.3)	16 (31.4)		19 (36.5)	16 (30.8)		15 (28.8)	20 (39.2)	
Child-Pugh class, N (%)			1.00			0.62			0.62			1.00			0.06
A	36 (97.3)	38 (95.0)		48 (94.1)	50 (98.0)		48 (94.1)	50 (98.0)		50 (96.2)	50 (96.2)		52 (100)	47 (92.2)	
B	1 (2.7)	2 (5.0)		3 (5.9)	1 (2.0)		3 (5.9)	1 (2.0)		2 (3.8)	2 (3.8)		0 (0.0)	4 (7.8)	
AFP, N (%)			0.04			0.05			0.32			<.00			0.17
< 200 IU/mL	14 (38.9)	25 (64.1)		20 (39.2)	30 (60.0)		22 (44.0)	28 (54.9)		0 (0.0)	52 (100)		29 (56.9)	21 (42.0)	
≥ 200 IU/mL	22 (61.1)	14 (35.9)		31 (60.8)	20 (40.0)		28 (56.0)	23 (45.1)	0.3220	52 (100)	0 (0.0)		22 (43.1)	29 (58.0)	
Prior systemic therapy, N (%)			0.77			0.11			0.60			0.80			0.61
≤ 60 days	6 (16.2)	8 (20.0)		12 (23.5)	5 (9.8)		7 (13.7)	10 (19.6)		10 (19.2)	8 (15.4)		8 (15.4)	10 (19.6)	
> 60 days	31 (83.8)	32 (80.0)		39 (76.5)	46 (90.2)		44 (86.3)	41 (80.4)		42 (80.8)	44 (84.6)		44 (84.6)	41 (80.4)	
Hepatitis virus history, N (%)			0.18			0.10			0.13			0.01			0.09
HBV positive	7 (18.9)	6 (15.0)		7 (13.7)	9 (17.6)		6 (11.8)	10 (19.6)		12 (23.1)	4 (7.7)		9 (17.3)	7 (13.7)	
HCV positive	18 (48.6)	12 (30.0)		26 (51.0)	16 (31.4)		24 (47.1)	18 (35.3)		24 (46.2)	19 (36.5)		19 (36.5)	24 (47.1)	
HBV and HCV negative	11 (29.7)	21 (52.5)		15 (29.4)	25 (49.0)		21 (41.2)	19 (37.3)		16 (30.8)	24 (46.2)		24 (46.2)	16 (31.4)	

Abbreviations: MET, the receptor tyrosine kinase encoded by the homonymous *MNNG-HOS* transforming gene; HGF, hepatocyte growth factor; AFP, alpha-fetoprotein; VEGF, vascular endothelial growth factor; ECOG PS, Eastern cooperative oncology group performance status; HBV, hepatitis B virus; HCV, hepatitis C virus.

When distributing patients by H-score, the difference between MET-High and -Low patients was clear, with median H-score of 175 in MET-High and 40 in MET-Low patients. Approximately half the patients (48%) were found to be MET-High. Biopsy date was known for 72 samples. When samples were analyzed with respect to prior therapy, the chance of being MET-High was 40% if the biopsy was obtained before sorafenib (*N* = 55), and 82% if obtained after sorafenib (*N* = 17; Table 2).

**Table 2:** Tumor MET status by prior therapy.

	MET-High	MET-Low
Overall* (N=77)	37 (48%)	40 (52%)
Median time on sorafenib	6.1 months	4.6 months
Samples taken before sorafenib (N=55)	22 (40%)	33 (60%)
At surgery (N=9)	0 (0%)	9 (100%)
Before TACE (N=18)	6 (33%)	12 (67%)
After TACE (N=7)	6 (86%)	1 (14%)
Samples taken after sorafenib (N=17)	14 (82%)^a^	3 (18%)^b^
Median H-score (range: 0-300)	175 (120-300)	40 (0-125)

Abbreviations: MET, the receptor tyrosine kinase encoded by the homonymous *MNNG-HOS* transforming gene; TACE, transarterial chemoembolization.

* sampling date available for 72 of the 77 patients.

a including 6 patients treated with TACE;

b including 1 patient treated with TACE.

**Figure 1:**
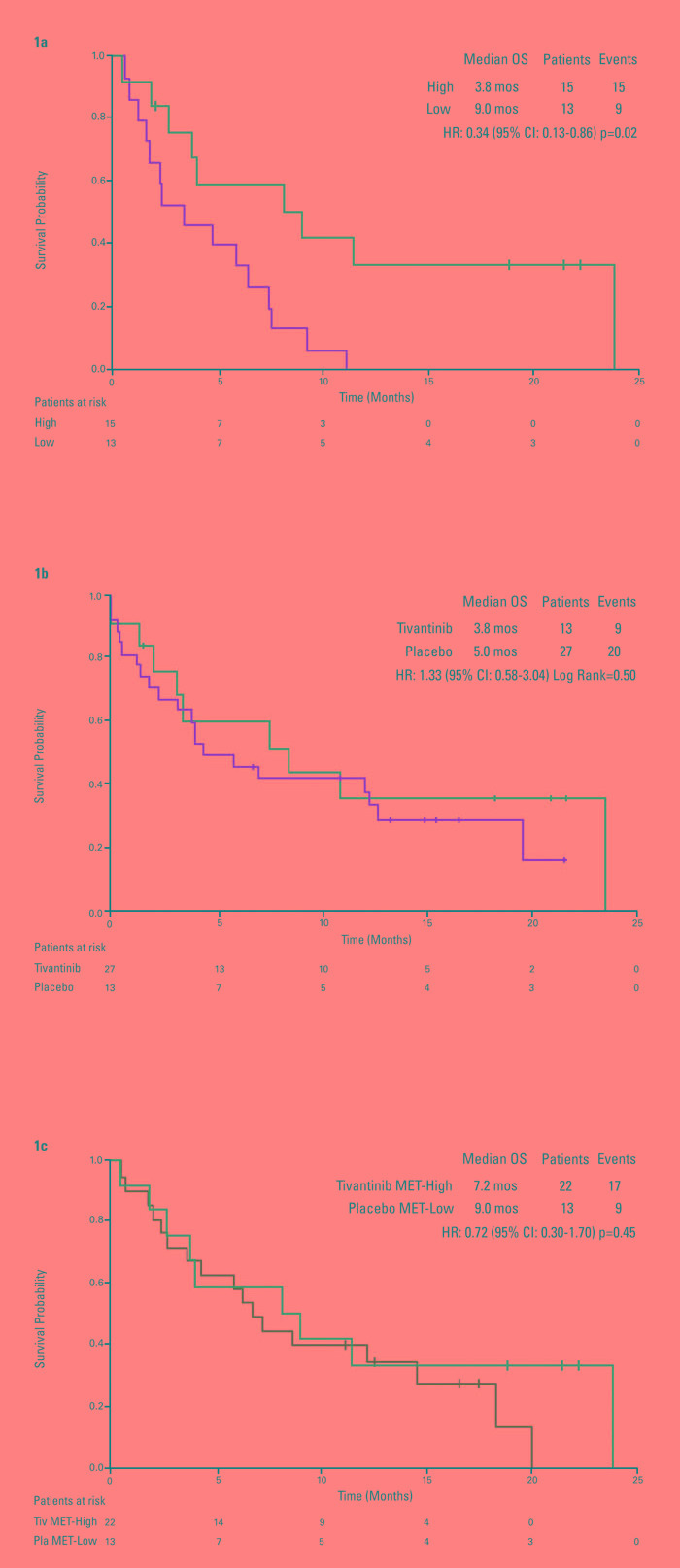
Kaplan-Meier analysis of overall survival by tumor MET **1a.** placebo patients by tumor MET status*. **1b.** tivantinib *versus* placebo in MET-Low patients. **1c.** placebo MET-Low *versus* tivantinib MET-High patients. *Figure reprinted and adapted from (Rimassa et al, Hepat Oncol 2014), with permission from Future Medicine.

As previously published, tumor MET was found to have a prognostic role. For patients receiving placebo, survival was longer for MET-Low patients than MET-High patients (HR 0.34, *p* = 0.02; Figure 1a). MET-High expression correlated with tivantinib efficacy (overall survival (OS): hazard ratio [HR] 0.38, 95% confidence intervals (CI) 0.18-0.81, *p* = 0.01). Tivantinib was ineffective in patients with MET-Low tumors (Figure 1b) [8].

Interestingly, no significant difference was found between survival of MET-Low patients on placebo and MET-High patients on tivantinib (HR 0.72, 95% CI, 0.30-1.70, *p* = 0.45), suggesting that tivantinib may offset the negative prognostic impact of high MET expression (Figure 1c).

The test for interaction between treatment and MET status showed a statistical significance at an alpha level of 0.05 for OS (*p* = 0.04). Correlation between MET and other baseline characteristics was not detected except for a slight overlap between MET-High patients and patients with a baseline AFP higher than the median.

### Circulating MET

The prognostic and predictive values of circulating MET were studied in 102 patients (*N* = 68 on tivantinib, *N* = 34 on placebo). The median MET concentration at baseline was 13.26 ng/mL, with a range of 1.29-49.8 ng/mL. Eighty-six patients (*N* = 56 on tivantinib, *N* = 30 on placebo) who were evaluable for survival were also evaluable for changes in circulating MET over the course of the study. Baseline characteristics were generally balanced between groups, except for higher prevalence of hepatitis C virus (HCV) infection (56.9% *versus* 33.3%, *p* = 0.03) in the circulating MET-High compared to the MET-Low group (Table 1). No correlation was found between circulating MET and tumor MET status.

**Figure 2:**
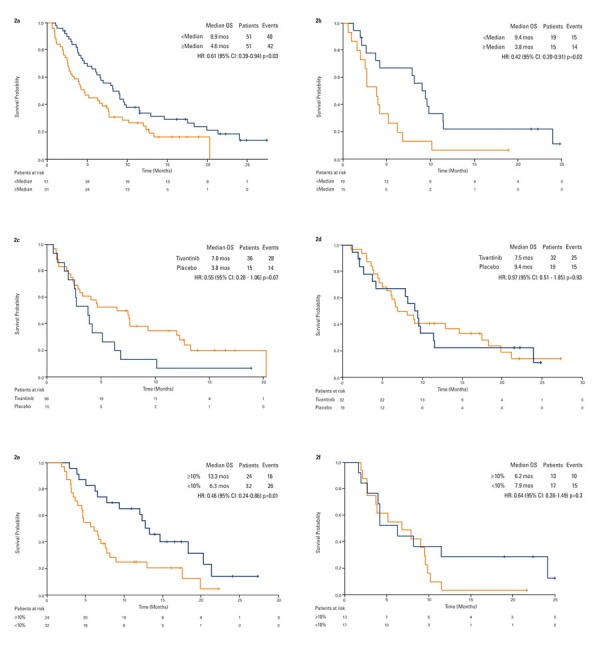
Kaplan-Meier analysis of overall survival by circulating MET **2a.** overall population by baseline circulating MET status. **2b.** placebo patients by baseline circulating MET status. **2c.** tivantinib *versus* placebo in circulating MET-High patients. **2d.** tivantinib *versus* placebo in circulating MET-Low patients. **2e.** tivantinib patients by best change in circulating MET. **2f**: placebo patients by best change in circulating MET.

Overall, circulating MET-Low patients survived longer than MET-High patients (HR 0.61, 95% CI, 0.39-0.94, *p* = 0.03; Figure 2a). When analyzing such results by treatment arm, a significant difference in terms of OS was observed in patients on placebo: 3.8 months in 15 circulating MET-High and 9.4 months in 19 circulating MET-Low patients (HR 0.42, 95% CI, 0.20-0.91, *p* = 0.02; Figure 2b). Survival in circulating MET-High patients was 7.0 months on tivantinib (*N* = 36) and 3.8 months on placebo (*N* = 15), (HR 0.55, 95% CI, 0.28-1.06, *p* = 0.07). The OS in circulating MET-Low patients was 7.5 months on tivantinib (N = 32) and 9.4 months on placebo (*N* = 19), (HR 0.97, 95% CI, 0.51-1.85, *p* = 0.93; Figures 2c and 2d). However, the test for interaction between circulating MET values and treatment was not significant.

Patients on tivantinib whose circulating MET dropped by at least 10% survived longer than patients with no pharmacodynamic response, with a median OS of 13.3 and 6.3 months, respectively (HR 0.46, 95% CI, 0.24-0.86, *p* = 0.01; Figure 2e). Applying the landmark method in which no subject died before 8 weeks, results remained unchanged (HR 0.46, 95% CI, 0.24-0.86, *p* = 0.01). Such an advantage was evident by week 8 of therapy (OS 13.3 months in 21 patients with MET reduction, 6.5 months in 35 patients with no or minimal MET reduction; HR 0.44, 95% CI, 0.23-0.86, *p* = 0.01). No such trend was observed in patients receiving placebo (Figure 2f). Furthermore, in patients with stable disease at the first tumor assessment, median change from baseline MET was −37.9% (std = 92.3, mean = 6.9, min = −75.8, max = 301.1) on tivantinib, +18.4% (std = 112.4, mean = 41.7, min = −89.0, max = 242.1) on placebo.

### Circulating HGF

The prognostic and predictive values of circulating HGF were evaluated in 102 patients (*N* = 68 on tivantinib, *N* = 34 on placebo). The baseline median HGF concentration was 2307 pg/mL, with range of 421-58080 pg/mL. Eighty-six patients (*N* = 56 on tivantinib, *N* = 30 on placebo) who were evaluable for survival were also evaluable for changes in circulating HGF over the course of the study. Baseline characteristics were generally balanced between groups (Table 1). No correlation was found between baseline HGF and circulating or tumor MET.

**Figure 3:**
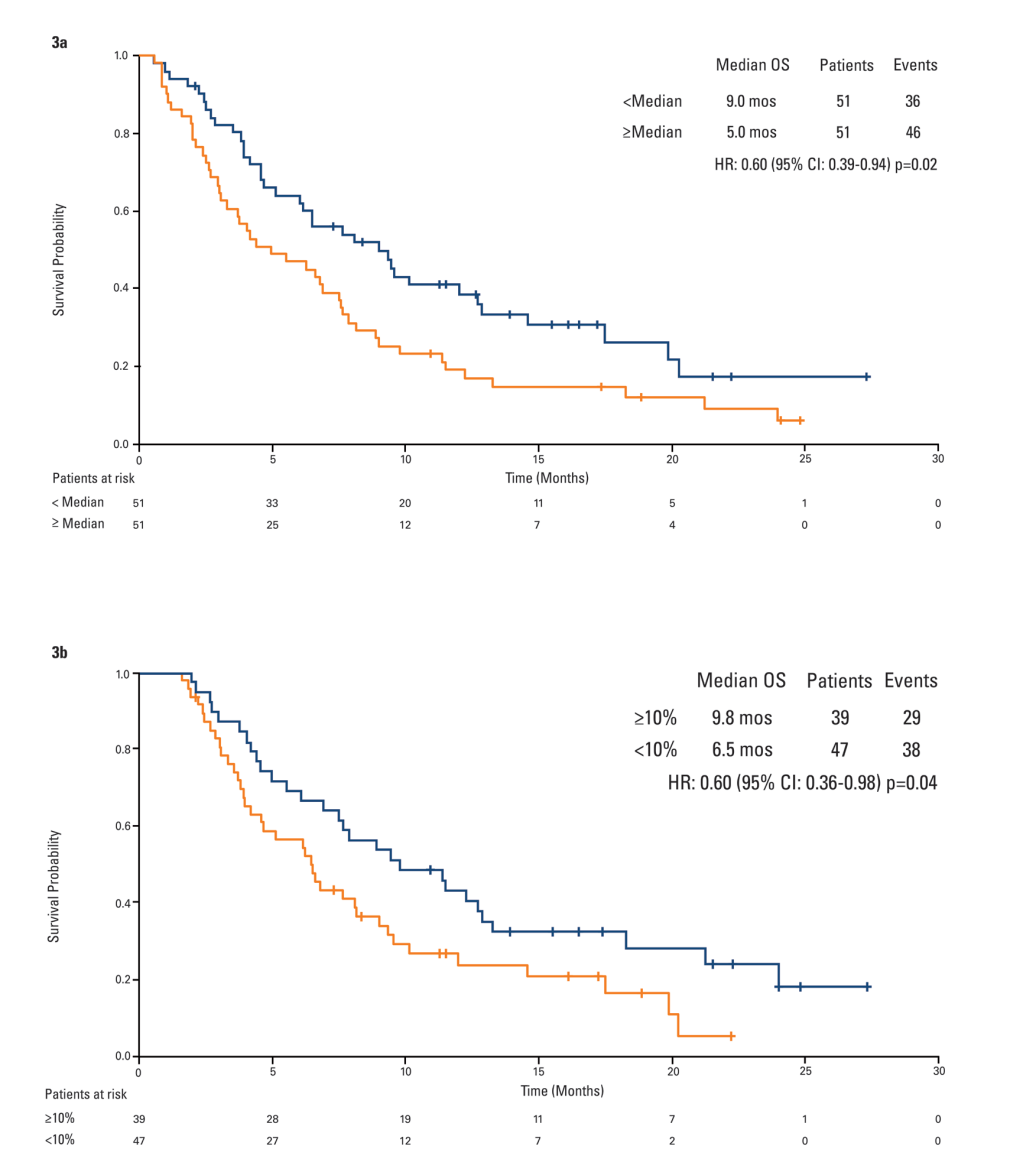
Kaplan-Meier analysis of overall survival by circulating HGF **3a.** overall population by baseline circulating HGF status. **3b.** overall population by best change in circulating HGF.

Patients with a baseline HGF lower than the median survived longer than patients with a higher baseline HGF regardless of the therapy (9.0 months *versus* 5.0 months; HR 0.60, 95% CI, 0.39-0.94, *p* = 0.02; Figure 3a). When analyzing such results by treatment arm, a significant difference in terms of OS was observed for patients on tivantinib (5.2 months in 30 HGF-High patients, 9.3 months in 38 HGF-Low patients; HR 0.57, 95% CI, 0.33-0.98, *p* = 0.04) but not for patients on placebo (4.2 months in 21 HGF-High, 9.0 months in 13 HGF-Low patients, HR 0.80, 95% CI, 0.37-1.73, *p* = 0.56). Furthermore, the test for interaction shows no correlation between HGF and OS benefit with tivantinib.

Patients with a reduction over time by at least 10% in circulating HGF survived longer than patients with no or minimal reduction (9.8 months *versus* 6.5 months; HR 0.60, 95% CI, 0.36-0.98, *p* = 0.04; Figure 3b). Applying the landmark method in which 1 subject who died before 8 weeks was removed from the analysis, results remained substantially unmodified (HR 0.61, 95% CI, 0.37-1.00, *p* = 0.05). No difference in OS was observed when comparing patients with and without HGF reduction on tivantinib (HR 0.68, 95% CI, 0.37-1.26, *p* = 0.22) or placebo (HR 0.48, 95% CI, 0.20-1.10, p = 0.08). In patients with stable disease past the first tumor assessment, median change from baseline HGF was +28.2% (std = 37.5, mean = 25.0, min = −96.6, max = 80.1) regardless of treatment.

Analysis of outcomes by baseline HGF as well as by changes in HGF over the course of the study suggests that HGF may correlate with outcome but may not be predictive of benefit from tivantinib.

### Circulating AFP

Circulating AFP was measured at baseline in 104 patients, all evaluable for OS (68 on tivantinib, 36 on placebo). The baseline median AFP concentration was 186 IU/mL, with range of 1.5-440008 IU/mL. Baseline characteristics were generally balanced between groups (Table 1). Correlations between AFP and tumor/circulating MET were not meaningful.

A trend towards better outcome was seen in patients with a baseline AFP lower than the median, regardless of treatment allocation (AFP-Low: *N* = 52, median OS 7.8 months; AFP-High: *N* = 52, median OS 5.0 months; HR 0.75, 95% CI, 0.48-1.15, *p* = 0.18). To understand if higher AFP values have a greater prognostic impact, an additional analysis was performed using an arbitrary cut-off value equal to the AFP 75^th^ percentile (third quartile (Q3); 3507.50 IU/mL) resulting in longer OS for patients with baseline values lower than Q3 (AFP-<Q3: *N* = 78, median OS 7.9 months; AFP-≥Q3: *N* = 26, median OS 3.0 months; HR 0.36, 95% CI, 0.22-0.58, *p* < 0.0001).

Only 43 patients (31 on tivantinib, 12 on placebo) had baseline values ≥20 IU/mL, had samples at multiple time points, and were evaluable for OS; therefore, no analysis was performed on AFP changes over time.

Survival of patients on tivantinib *versus* placebo by any AFP status was comparable, with the test for interaction resulting non-significant.

### Circulating VEGF

Circulating VEGF was measured in 103 patients also evaluable for OS (69 on tivantinib, 34 on placebo). Median baseline circulating VEGF was 160 pg/mL. Baseline characteristics were generally balanced between groups (Table 1). Survival was 9.0 months in patients with VEGF-Low (*N* = 51), 5.0 months in patients with VEGF-High (*N* = 52) (HR 0.69, 95% CI, 0.45-1.06, *p* = 0.09); no trend was observed by treatment arm. Survival of patients with a VEGF reduction over time by at least 10% tended to be longer than survival of patients with no or minimal reduction (8.1 months *versus* 6.8 months; HR 0.78, 95% CI, 0.48-1.26, *p* = 0.31), regardless of treatment.

## DISCUSSION

Prognostic and predictive biomarkers are integral to fully understand the disease and to establish efficacious treatments in the era of targeted therapies. In HCC, retrospective analyses to identify biomarkers have proven challenging due to molecular heterogeneity, clonal evolution driving drug resistance [34], and the analyses being conducted in uncontrolled studies.

The analyses presented here were conducted on data from the ARQ 197-215 study of tivantinib in second-line HCC, the first placebo-controlled randomized study to evaluate a tumor biomarker and to identify biomarkers prognostic and predictive of outcome. Although numbers for some of the analyzed subgroups were small, the population was homogeneous, received sorafenib as prior systemic therapy, and the analyses had been defined, before unblinding, in the statistical analysis plan.

The prognostic role of tumor MET has been previously published, with patients on placebo surviving 3.8 months if MET-High, and 9.0 months if MET-Low [8]. In the current analysis, we observed a dramatic difference in MET status between biopsies taken before and after sorafenib treatment. This is in line with literature suggesting that high MET expression correlates with hypoxia, resistance to anti-angiogenic therapies, and poor prognosis in HCC [10-12, 35], and further supports the prognostic role of MET. A correlation between MET status and transarterial chemoembolization (TACE), which also causes hypoxia, could not be assessed due to the small number of TACE-treated patients in this study (Table 2). Our results, confirmed by data from the larger METIV-HCC study, suggest that biopsies taken after sorafenib can more reliably assess the prognostic status of MET in second-line HCC patients [36]. Moreover, the weight of MET-Low as a positive prognostic factor may have been underestimated in the ARQ 197-215 study because MET staining was largely assessed on pre-sorafenib samples. This also emphasizes the need to biopsy HCC patients following sorafenib treatment in order to succeed in biomarker research, and to cautiously consider therapeutic options based on life expectancy.

In addition, tumor MET was found to be predictive of outcome in tivantinib-treated patients with a positive interaction test. Comparison of survival curves suggested that tivantinib offsets the negative impact of MET, making survival of the MET-High treated patients similar to the MET-Low population. In the ARQ 197-215 trial, despite the high incidence of MET-High status after sorafenib, the lack of efficacy of tivantinib in MET-Low patients combined with their good prognosis on placebo did not allow the overall unselected population to gain a survival benefit from tivantinib. The above considerations rely on IHC, a technique often criticized for its subjectivity. Previously reported studies have employed a relatively low cut-off to define MET positivity. As an example, studies with rilotumumab defined as MET-High any patient with at least 25% of tumor cells staining with any intensity, converting to a minimum H-score of 25 for MET-High patients [37]. To avoid technique-specific biases, studies with tivantinib employed a more conservative reading criterion, considering MET-High only samples showing both high and diffuse intensity of staining and excluding any borderline or slightly positive cases, resulting in a minimum H-score of 120 for MET-High patients. Inter-study variability in defining MET-High status may have an impact on the assessment of its prognostic and predictive value, and employing a rigorous testing standard allows to select the most appropriate population for anti-MET therapies.

Publications on circulating biomarkers for second-line HCC are very limited, the most significant recent findings being the possible predictive role of AFP for ramucirumab and the potential correlations between biomarkers and prognosis, etiology and ethnicity found in the EVOLVE-1 trial [38, 39].

In the ARQ 197-215 study, the analyses of efficacy endpoints by circulating biomarkers suggested a prognostic value for circulating MET and HGF, and a trend towards a predictive role only for circulating MET. The number of patients did not allow a multivariate analysis to exclude the influence of tumor MET on circulating factors, therefore such results are to be confirmed in larger studies. On the other hand, findings with HGF were in line with what was reported for first-line patients in the biomarker analysis from the SHARP study [40]. In HCC patients pre-treated with sorafenib, AFP was ascribed a prognostic role, as confirmed by the BRISK-PS and REACH studies [38, 41]. However, in the ARQ 197-215 study, circulating MET and HGF provided clearer results than AFP, with a similar number of evaluable patients. Median values were sufficient to give a statistical survival advantage to patients with low levels of either MET or HGF, while lower than median AFP only trended towards a better outcome. Similar results (data not shown) were obtained with the 200 and 400 IU/mL cut-off values used in other trials [38, 42]. Further analysis correlated higher AFP baseline values with even shorter survival.

The pharmacodynamic role of MET was suggested by two observations in patients on tivantinib but not on placebo: patients who had a reduction over time in circulating MET survived longer, as did patients whose tumor was stable at first scan and whose circulating MET level was reduced from baseline. The observed correlation between HGF changes and outcome is in line with data demonstrating HGF is produced by active HCC, and therefore non-responsive tumors continue to release it into the bloodstream [43].

The trend towards a role for circulating MET in predicting benefit from tivantinib supports the overall use of MET as a biomarker in tivantinib studies. However, unlike the reported results from the 77 tumor samples tested for MET, analysis of OS in relation to baseline circulating MET and treatment in the 102 patients did not reveal a statistical interaction. Therefore, the power of predicting who will respond to tivantinib is greater for tumor than circulating MET, and provides rationale to use only the former to select patients for clinical trials with tivantinib. This is consistent with results previously reported in randomized, placebo-controlled studies in colorectal, NSCLC, and prostate cancer [27, 28, 30].

Correlations between biomarkers remain exploratory, especially considering the study sample size. Correlations with tumor MET are difficult to assess because many MET-Low results were determined on biopsies performed before treatment with sorafenib. Correlation between MET and HGF was not expected because, as with other receptors, MET could be activated independently [13, 44]. Correlation between MET and AFP could not be clearly assessed, since the number of patients in whom both were high or low was small and either factor could predict aggressive disease independently.

Although the number of patients in the ARQ 197-215 was relatively small, this remains the only randomized clinical trial to date that prospectively evaluated a tumor biomarker, and is among the few that evaluated multiple circulating biomarkers in HCC. Biological selection of patients remains crucial for the development of targeted therapies in this population, as demonstrated by the general lack of success of phase III studies with unselected HCC patients, except for the recent regorafenib study [45]. Only recently the REACH study, although negative in the overall population, identified AFP as a possible biomarker for patient selection for treatment with ramucirumab [38].

In conclusion, our analysis suggests baseline circulating MET and HGF as prognostic factors in second-line HCC, and tumor MET as a prognostic and predictive biomarker of HCC and tivantinib activity. Given the limitations intrinsic in a retrospective analysis from a phase II study, results need to be confirmed in larger trials. The ongoing METIV-HCC and JET-HCC phase III studies of tivantinib in the second-line setting (NCT01755767 and NCT02029157) enrolled only patients with tumor MET-High HCC. Such studies may clarify the role of MET, HGF, and AFP in this patient population.

## MATERIALS AND METHODS

### Tumor MET

MET expression was evaluated by IHC assay, on the most recent sample available, using the CONFIRM™ anti-total MET(SP44) antibody (catalog # 7904430, Ventana Medical Systems, Tucson, AZ), analyzed at an independent Clinical Laboratory Improvement Amendments (CLIA) certified laboratory by a board certified pathologist after randomization and prior to study un-blinding. MET-High was defined as a majority (≥50%) of tumor cells with moderate or strong (2+ or 3+) staining intensity per ArQule's protocol. A negative control slide from each patient sample was run as well as positive control from colon adenocarcinoma ensuring that proper antibody and reagent dispensing took place. Additionally, results of MET testing were reported as an H-score, calculated by multiplying the percentage of cells staining by the intensity of the stain [46].

### Circulating biomarkers

Serum biomarker concentrations were measured by ICON Central Labs, Farmingdale, NY, using commercial materials.

### MET/HGF/VEGF

MET, HGF and VEGF serum samples were collected before the first dose on cycle 1 day 1, and post dose every 4 weeks thereafter, and at treatment end. One blood collection tube was used for MET/HGF testing and another for VEGF testing.

For circulating MET measurement, Kamiya Biomedical K-ASSAY Human MET ELISA kit (catalog # KT-444), Automated ELISA System (Dynex Technologies), and the Spectramax reader (Molecular Devices) were used. The Quantikine Human HGF Immunoassay (R&D Systems, Minneapolis, MN, catalog # SHG00) was used to measure HGF levels in serum using the Spectramax reader (Molecular Devices). For VEGF, QuantiGlo Human VEGF immunoassay kit from R&D (catalog # SVE00) using the Spectramax Plus 384 reader was used.

### AFP

AFP serum samples were collected at screening/pre-study, and every 8 weeks after randomization, as well as at the end of treatment. AFP levels were tested using automated analyzer Immulite 2000 (kits supplied by Siemens Diagnostics, Los Angeles, CA).

### Statistical analysis

All statistical analyses were performed using SAS statistical software. Biomarker baseline values and their changes over the course of treatment were dichotomized and analyzed with respect to OS probability. Median OS and the 95% CI were estimated using the Kaplan-Meier method and the logrank test was used to compare the survival distributions. The Cox regression model was used to obtain the HR. The interaction between biomarkers and the treatment was evaluated using the Cox proportional hazards model with an interaction term. Multivariate analysis was not attempted due to the small sample size of available biopsies for tumor MET biomarker assessment. The correlation between the biomarkers was assessed using Fisher's exact test. The median concentrations of the serum baseline biomarkers were used as the cut-off values to differentiate the respective Low and High groups for the study of prognostic and predictive effects. However, in the case of AFP, an additional arbitrary cut-off was set at the 75^th^ percentile. As for the changes in biomarker concentrations, the best change (highest reduction or the lowest increase) during the treatment period for each patient was calculated as the percent change from baseline, and 10% was arbitrarily chosen as cut-off value. Survival analysis by biomarker reduction was also performed restricting the sample to patients who survived to a pre-specified arbitrary landmark time of 8 weeks after the date treatment was started. This latter analysis was specifically undertaken to eliminate guarantee-time bias and was proposed by others in similar settings [47, 48].
